# Microscopic modeling of charge transport in sensing proteins

**DOI:** 10.1186/1556-276X-7-340

**Published:** 2012-06-22

**Authors:** Lino Reggiani, Jean-Francois Millithaler, Cecilia Pennetta

**Affiliations:** 1 Dipartimento di Ingegneria dell’Innovazione and CNISM, Università del Salento, Via Arnesano, Lecce, 73100, Italy; 2 Dipartimento di Matematica e Fisica Ennio De Giorgi and CNISM, Università del Salento, Via Arnesano, Lecce, 73100, Italy

**Keywords:** Impedance network, Bacteriorhodopsin, Olfactory receptors, Conformational change, Nano-biosensors

## Abstract

Sensing proteins (receptors) are nanostructures that exhibit very complex behaviors (ions pumping, conformational change, reaction catalysis, etc). They are constituted by a specific sequence of amino acids within a codified spatial organization. The functioning of these macromolecules is intrinsically connected with their spatial structure, which modifications are normally associated with their biological function. With the advance of nanotechnology, the investigation of the electrical properties of receptors has emerged as a demanding issue. Beside the fundamental interest, the possibility to exploit the electrical properties for the development of bioelectronic devices of new generations has attracted major interest. From the experimental side, we investigate three complementary kinds of measurements: (1) current-voltage (I-V) measurements in nanometric layers sandwiched between macroscopic contacts, (2) I-V measurements within an AFM environment in nanometric monolayers deposited on a conducting substrate, and (3) electrochemical impedance spectroscopy measurements on appropriate monolayers of self-assembled samples. From the theoretical side, a microscopic interpretation of these experiments is still a challenging issue. This paper reviews recent theoretical results carried out within the European project, Bioelectronic Olfactory Neuron Device, which provides a first quantitative interpretation of charge transport experiments exploiting static and dynamic electrical properties of several receptors. To this purpose, we have developed an impedance network protein analogue (INPA) which considers the interaction between neighboring amino acids within a given radius as responsible of charge transfer throughout the protein. The conformational change, due to the sensing action produced by the capture of the ligand (photon, odour), induces a modification of the spatial structure and, thus, of the electrical properties of the receptor. By a scaling procedure, the electrical change of the receptor when passing from the native to the active state is used to interpret the macroscopic measurement obtained within different methods. The developed INPA model is found to be very promising for a better understanding of the role of receptor topology in the mechanism responsible of charge transfer. Present results point favorably to the development of a new generation of nano-biosensors within the lab-on-chip strategy.

## Review

Receptors are proteins of relevant interest because of the fundamental role they play in living environments at a cellular level [1,2]. At present, the best known (and studied) are bacteriorhodopsin (bR), a light sensitive protein whose action is related to a proton pump, and some of the G protein-coupled receptors, a large class of proteins that are sensitive to the light or more generally to the capture of single or a few specific molecules (ligands). The activation of a receptor is driven by the capture of an external ligand [3] and starts with a conformational change followed by a biological chain of events finally detected by the brain. The intriguing question is whether this biological chain of detection could be replaced by an electrical chain monitored by the change of an electrical property of the single protein induced by the change of conformation. To answer this question, the investigation of the electrical properties of a protein is the essential step.

To this purpose, we mention recent experiments on the current-voltage (I-V) characterization of bR monolayers sandwiched between metallic contacts [4,5]. Measurements were carried out in dark and when the sample was irradiated with a green light to which bR is sensitive. At increasing voltages, the current was found to exhibit a superlinear behavior which is common to both the illumination conditions. Furthermore, for each voltage value, the current is found to be significantly enhanced up to about a factor of three by the presence of light. These results, besides confirming the very low electrical conductivity of bR, suggest an underlying tunneling mechanism of charge transport [6,7]. On the other hand (most interesting for technological applications), following the I-V response, it is possible to monitor the protein activation, i.e., the conformational change. By implementing an atomic force microscope (AFM) technique, I-V measurements on bR were extended to higher applied voltages (near to about 10 V) where a cross over from direct to injection (or Fowler Nordheim [6]) tunneling regime was evident [8,9]. On the same subject, recent electrical experiments have been performed on the rat olfactory receptor (OR) OR I7 and on the human OR 17-40 properly deposited on a functionalized gold substrate with the technique of molecular self-assembly. These measurements [10,11] showed the possibility to monitor the sensing action of the protein and, in turn, their conformational change by means of the modification of the electrochemical impedance spectrum in the presence of a controlled flux of specific odorants (heptanal, octanal). From a theoretical side, a microscopic interpretation of all these experiments is still a challenging issue.

The aim of the present work is to report on a microscopic model, recently developed by the authors [3,12-16], which is able to interpret the electrical properties of a single protein and their modifications due to the sensing action in terms of the change of the protein tertiary structure. By implementing an impedance network protein analogue (INPA), the theoretical model is henceforth called INPA model. To this scope, we further included in the model a sequential tunneling mechanism [14-16] able to account for the superlinear I-V characteristics experimentally evidenced in bR and similar proteins at increasing applied voltages. Then, the model is validated on available experiments and its predictability discussed in the perspective of further experimental confirmations.

### Model and materials

The INPA model is briefly summarized below. Each protein is associated with a topological network (graph) consisting of nodes and links. Once the tertiary structure of the protein is given by the protein database (PDB) [17] or similarly, the graph reproduces its main features. To this purpose, the nodes are in correspondence with the amino acids, taken as single interacting centers, and their position coincides with the related C_*α*_ atom. Each couple of nodes is connected with a link when their distance is less than an assigned interacting radius, R_*C*_. In this way, the protein network analogue becomes a set of intercrossing spheres of radius R_*C*_. In principle, all the values of R_*C*_ from zero to tens of nanometers (the protein size) are possible. Actually, this value depends on the kind of interaction under consideration [12,18-20]. In the present case, as interaction, we consider the transfer of charge. Accordingly, R_*C*_ is taken in the range 5 to 15 Å, as will be detailed in the following. Finally, the network gives a skeleton of the protein topology in a fixed conformation.

**Figure 1:**
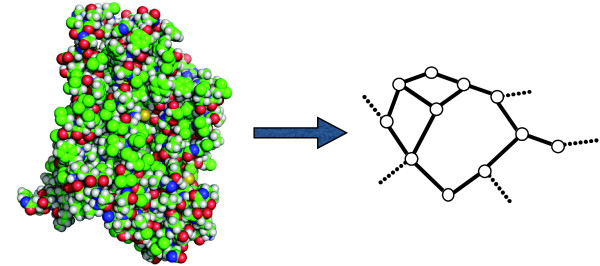
**Schematic of the INPA model.** The left-hand side shows the all-atom picture of a protein. The right-hand side is the partial sketch of the equivalent network obtained for a given value of the interaction radius, *R*_*C*_ (see text).

**Figure 2:**
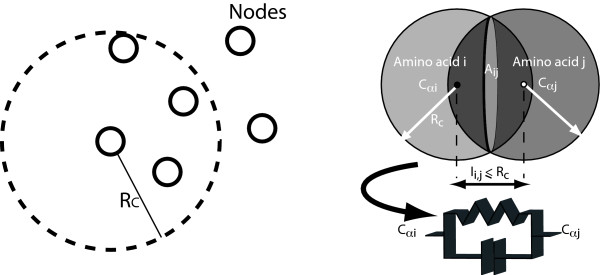
**Schematic of the elemental impedance.** The left-hand side is the selection of the interacting amino acids (nodes) within a distance smaller than the interaction radius, *R*_*C*_. The right-hand side is the schematic view of the elementary RC impedance replacing one link between the *α*-carbon atoms of the *i*-th and *j*-th amino acids when their distance *l*_*i*,*j*_ is smaller than *R*_*C*_.

Figure 1 shows how the full atomic representation of a protein transforms into the sketch of a possible graph. The graph turns into an impedance network when an elemental impedance is associated with each link. In the present case, following the experimental outcomes, elementary impedance consisting of a resistance R parallelly connected with a capacitance C as shown in Figure 2. The elemental impedance between the *i*-th and *j*-th nodes is taken as [21]:

(1) $$Z_{i,j}=\frac{l_{i,j}}{A_{i,j}}\frac{1}{\left(\rho^{-1}+i\varepsilon_{i,j}\;\varepsilon_{0}\omega \right)},$$

where $A_{i,j}=\Pi (R_{C}^{2}-l_{i,j}^{2}/4)$, is the cross-sectional area between the spheres centered on the *ij* nodes, respectively; *l*_*i*,*j*_ is the distance between these centers; *ρ*is the resistivity taken to be the same for every amino acid with the indicative value, *ρ* = 10^10^*Ω*m (here, the microscopic mechanism responsible of charge transfer is not specified); $i=\sqrt{-1}$ is the imaginary unit; *ε*_0_ is the vacuum permittivity; and *ω*is the circular frequency of the applied harmonic voltage. The relative dielectric constant pertaining to the couple of *ij* amino acids, *ε*_*i*,*j*_, is expressed in terms of the intrinsic polarizability of the *ij* amino acids [21].

By positioning the input and output electrical contacts on the first and last node, respectively for a given applied bias, the network is solved within a linear Kirchhoff scheme, and its global impedance spectrum, *Z*(*ω*), is calculated in the standard frequency range 0.1 to 10^5^ Hz. By construction, besides the small-signal dynamic response, this network produces a parameter dependent static I-V characteristic determined as

(2) V=Z(0)I

However, since the purpose is to monitor the impedance variation of the receptor due to the conformational change, the relative change of impedance, rather than its absolute value, is taken as the relevant output of the modeling. The spatial distribution of the obtained network is found to be, in general, highly irregular. Accordingly, it describes the protein topology only when the tree structure is dominant, i.e., when the value of *R*_*C*_ is sufficiently small so that the links between nearest neighbors predominate. We remark that the tree structure might not suffice to reproduce the global functioning of the protein. Thus, it is necessary to find an optimal value of *R*_*C*_ which can reproduce both the structure and the function of the protein [12,13,20]. From one hand, the *R*_*C*_ value should be greater than the mean distance between contiguous C_*α*_ atoms, say 3.8 Å; otherwise, the network appears to be not connected, and the value of the single protein impedance tends to be infinite. From another hand, for very large values of *R*_*C*_, say 80 Å, each node will link all the others and the network is no longer descriptive of the protein topology. In other words, for values of *R*_*C*_ above about 80 Å the value of the impedance of the single protein no longer depends on *R*_*C*_. Accordingly, the best agreement between structure and function was obtained with values of *R*_*C*_ in the range 5 to 70 Å [12,13]. To determine nonlinear I-V characteristics, the model was implemented to include a sequential tunneling mechanism of charge transfer by means of a stochastic selection of low resistive links ruled by the voltage-dependent probability [14,15]:

(3) $$P_{i,j}=\text{exp}\left[-\frac{2l_{i,j}}{\hbar }\sqrt{2m(\Phi -eV_{i,j})}\right],$$

where *V*_*i*,*j*_ is the potential drop between the *i*-th and *j*-th node; *m* is an electron effective mass (taken as the bare one); *e* is the electron charge; *ℏ* is the reduced Planck constant; and *Φ* is the barrier height. The model has been further implemented to include also injection tunneling [16].

### Comparison between theory and experiments

As anticipated above, the INPA is able to interpret the behavior of the I-V characteristics carried out in bR with an *electrode-bilayer-electrode* structure and when the protein is illuminated or less by green light [4]. To this purpose, the model is applied to the PDB entries 2NTU (for the native state) and 2NTW (for the activated state of bR) [17]. The comparison between theory and experiments proceeds as follows. The theoretical model describes the I-V modifications of a single protein when it undergoes a conformational change. On the other hand, the experimental results are carried out on a macroscopic sample that contains a macroscopic number of proteins. To compare the results, we normalize the current value obtained for the single protein in its native state at 1 V to that of the experiment in dark. For the case of bR, the normalization corresponds to multiply the current of the single protein by a factor 10^8^ to 10^9^. The same normalization factor is used for the single protein in its activated state, which is then compared with the experimental value under green light.

**Figure 3:**
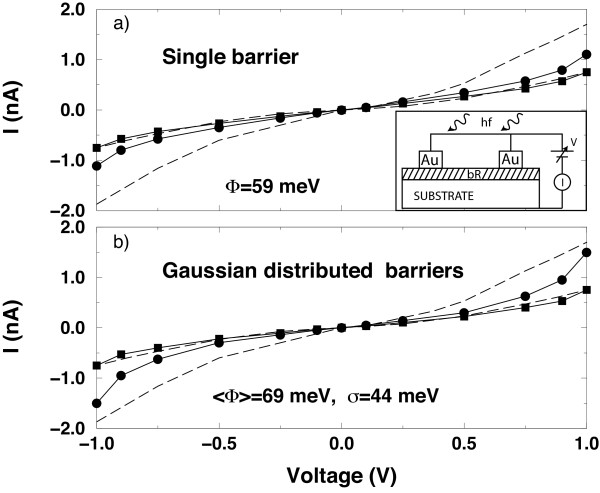
**Current-voltage characteristics on a large-area monolayer of bacteriorhodopsin.** Measured I-V characteristics of the native and activated state of bR are compared with theoretical calculations carried out using the 2NTU and 2NTW structures for the native and activated states, respectively. (**a**) - Full squares (circles) refer to calculations of the native (activated) state) carried out with *Φ* = 53 meV the same for each barrier; continuous curves are guides to the eyes, dashed curves refer to experiments carried out on a monolayer of bR as schematically shown in the inset of the figure [4]. (**b**) - The same as Figure 3 with calculations carried out with an average *Φ* = 69 meV Gaussian distributed with a variance *σ* = 44 meV. All the calculated currents are normalized to the experimental value of current in dark at 1 V [14].

Figure 3 reports the simulated results calculated for both the native and activated states in comparison with experiments. The trend evidenced by experiments is reproduced when considering an interacting radius of 6 Å and a single energy barrier between neighboring amino acids of 59 meV [14,15]. The agreement with experiments is improved when using a Gaussian distribution of barrier heights with average value of 69 meV and a standard deviation of 44 meV [14,15].

**Figure 4:**
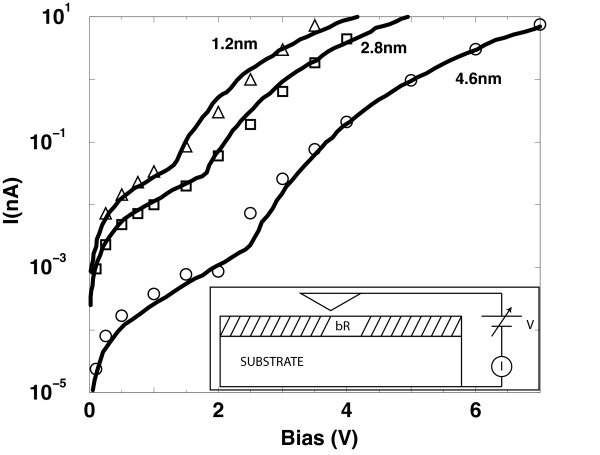
**Current voltage characteristics within an AFM technique on a monolayer of bacteriorhodopsin.** Measured I-V characteristics of the native state of bR are compared with theoretical calculations carried out using the 2NTU structure at the reported different indentations of the AFM tip. The AFM technique is sketched in the inset. The thick continuous lines show experimental data [8] and the symbols numerical simulations obtained with extended contacts including a leakage current [16].

Figure 4 compares the numerical and experimental data for the measurements carried out on bR within an AFM environment. The position of the contacts is then changed in order to reproduce the experiments obtained at different indentation of the AFM tip. At increasing depths of the extended contact, the net effect leads to a reduction in the number of amino acids involved in the electrical transport. As a consequence, we expect higher currents and a shift to lower potential values for the crossover between direct and injection tunneling regimes. Numerical calculations reported in Figure 4 confirm these expectations [16]. In particular, the flat contact that simulates the tip indentation was placed at the depths of 0.50 nm and 1.0 nm, respectively, from the top of the protein. For these depths, at increasing tip indentations, the experimental data exhibit a quantitative agreement with the modeling in the region of high voltages where the injection tunneling prevails. Even if the experimental indentation of the tip is greater for about a factor of three with respect to that of the simulations, we consider the agreement between theory and experiments to be satisfactory in view of the simplifications needed to convert the single protein calculation into the macroscopic value measured [16]. However, at low voltages, the results of the simulations underestimate for up to an order of magnitude the values of experiments. The disagreement at low voltages is overcome by assuming the existence of a leakage contribution, probably associated with the complexity of the contact regions constituted by trimers and lipids, with respect to the used model [22]. Accordingly, the single resistance associated with each link is replaced by the parallel of two resistances, one pertaining to the protein and the other to a more realistic modeling of the contact regions. Interestingly enough, the microscopic interpretation is obtained with a length-independent electron effective mass, contrary to the case of [8,9] where, to fit experiments, the carrier effective mass should increase over one order of magnitude at increasing the tip indentation. Both these features are consequences of the sequential tunneling mechanism [14-16] that replaces the single tunneling mechanisms of the metal-insulator-metal model previously used [8,9].

**Figure 5:**
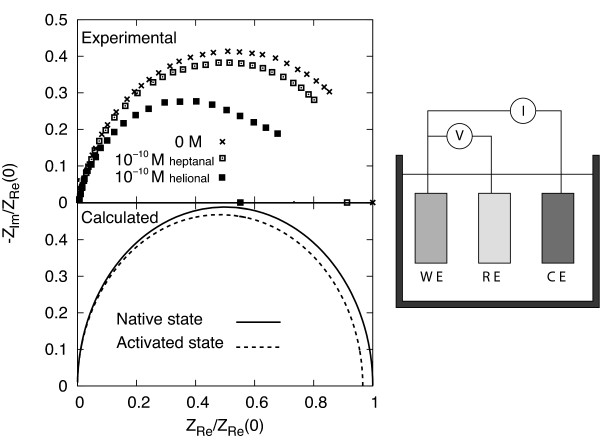
**EIS investigation on human OR 17-40.** Nyquist plot for the human OR 17-40 in the absence and in the presence of specific ligands (heptanal and helional molecules). The impedances are normalized to the static value of the native state *Z*_*real*_(0). In the upper figure, symbols report experiments carried out with a standard three electrode electrolytic cell sketched on the right-hand side part of the figure (with WE, working electrode; CE, counter electrode; RE, reference electrode). Here, crosses refer to the absence of an odorant and empty (full) squares to the presence of an heptanal (helional) odorants at concentration of 10^−10^*M*. In the lower part of the figure, lines report theoretical results with the continuous curve referring to the native state configuration with *R*_*C*_ = 70 Å and the dashed line referring to the active state configuration with *R*_*C*_ = 46 Å [3].

The impedance spectrum of a single olfactory receptor is investigated over a wide range of frequencies, and the results are reported by the associated Nyquist plot. This plot draws the negative imaginary part versus the real part of the global impedance within a given frequency range (typically from 1 mHz to 100 kHz as in experiments [23]). Figure 5 reports the Nyquist plots of human OR 17-40 with the impedance normalized to the static value of the native state. Symbols pertain to experiments, with crosses referring to the absence of a specific odorant, full (empty) squares pertain to the presence of the specific odorant helional (heptanal) at the concentrations reported in the figure. Curves pertain to theoretical results where the single protein is taken to be representative of the entire sample, and with continuous (dashed) lines referring to the native (active) state [3]. The agreement between theory and experiments is found to be satisfactory from a qualitative point of view and, apart for a underestimation of the maximum experimental value, acceptable from a quantitative point of view. We remark that the near-ideal semicircle shape of the experimental Nyquist plot is well reproduced, thus, confirming that the network impedance model behaves closely to a single RC circuit as expected by the presence of a rather uniform distribution of time constants associated with the different values of the resistance and capacitance of the links [13]. The scarcity of experimental data and the lack of a well-certified knowledge for the tertiary structures of the proteins under investigation lead us to consider these results as a first but significant step towards a microscopic modeling of the electrical properties of this olfactory receptor.

## Conclusions

The microscopic modeling of the electrical properties of a sensing protein is briefly reviewed with the objective of providing a unifying theoretical interpretation of available experiments. In this framework, protein tertiary structure plays a primary role. Indeed, by determining the network structure, its conformational change induces the network change and, consequently, the change of the electrical properties of the protein. The possibility to monitor the conformational change by means of electrical measurements is identified as the key point for a better knowledge of charge transport in biological materials and for exploiting new frontiers of application of sensing proteins. The qualitative and quantitative agreements between the numerical results and experiments pose the INPA, implemented to account for a sequential tunneling mechanism of charge transfer, as a physical plausible model to investigate the electrical properties in other proteins pertaining to or less to the receptor family.

## Competing interests

The authors declare that they have no competing interests.

## Authors’contributions

LR participated in the design of the study and drafted the manuscript. JFM participated in the design of the study and performed the statistical analysis. CP conceived of the study, and participated in its design and coordination. All authors read and approved the final manuscript.

## Authors’ information

LR is full professor in Physics of Matter at the Engineering Faculty of Salento University. JM received a PhD in micro-electronics from Montpellier University and has a post doc position at the Dipartimento di Ingegneria dell’ Innovazione of the Salento University. CP is an associate professor in Physics of Matter at the Science Faculty of Salento University.
